# Hospital Meetings, &c.

**Published:** 1901-10-26

**Authors:** 


### HOSPITAL MEETINGS, &c.

### THE QUEEN'S JUBILEE HOSPITAL.

On Tuesday the Lord Mayor opened some new buildings which have been erected in connection with the Queen's Jubilee Hospital, Richmond Road, Earl's Court. This extension consists of a small out-patient department, containing a waiting room, operating room, dispensary, two consulting rooms, and two other rooms. The building, which seems well suited for its purpose, has been erected upon a firm foundation so as to admit of extension upwards in the future, by the addition of an upper storey containing wards should the opportunity arise. The cost, with some few additions, furniture, instruments, etc., is estimated at about £2,500, of which sum £1,500 has already been subscribed. The erection of this new building has set free two small rooms in the house in which the work has hitherto been carried on, and it is stated that, by the conversion of these rooms into wards, ten additional beds for in-patients will be provided as soon as the necessary funds for their maintenance is forthcoming. In the speeches made on the occasion of the opening a good deal was made of the lack of hospital accommodation in the district, and of the necessity for making provision for the accidents which occur in the locality; and if it were the fact that this institution confined its operations to the sort of emergency work which was so well set forth as the plea for its existence, we should quite agree with much that was said on the subject. If, however, it is the fact that the funds collected for the purposes of this institution are being used, not for the maintenance of what may be termed an outpost hospital in which emergency cases which cannot bear removal to general hospitals may be received, but for the purpose of carrying on upon a small and comparatively inefficient scale the same sort of work as that which is being done at the general hospitals very much more effectively, then we need not hesitate to say that the continued existence of the institution is an injury and a drawback to hospital work in London. It may be that, by exciting local interest, the managers of the institution called the Queen's Jubilee Hospital are able to tap local sources of income which would not respond to any more general appeal, and if so we would be the last to say a word to damp the charity of the Earl's Court district; for charity is good and admirable in itself, even though it may not be applied with discretion. But when we find a small and purely local institution like this going outside its own proper field of work and endeavouring to draw towards itself, we might almost say to divert towards itself, a portion of that ever-flowing stream of charity which is excited by sympathy with the good work of the great hospitals, we cannot but feel that an injury is thereby being done to hospital work in London.

